# Mechanical and histological properties of native medial menisci compared to allograph medial menisci in the osteoarthritic knee

**DOI:** 10.3389/fbioe.2024.1364536

**Published:** 2024-04-19

**Authors:** Max Weiske, Bianca Riedel, Eva Johanna Kubosch, Hermann O. Mayr, Hagen Schmal, Michael Seidenstuecker

**Affiliations:** ^1^ G.E.R.N. Tissue Replacement, Regeneration & Neogenesis, Department of Orthopedics and Trauma Surgery, Medical Center-Albert-Ludwigs-University of Freiburg, Faculty of Medicine, Albert-Ludwigs-University of Freiburg, Freiburg, Germany; ^2^ Department of Orthopedics and Trauma Surgery, Medical Center-Albert-Ludwigs-University of Freiburg, Faculty of Medicine, Albert-Ludwigs-University of Freiburg, Freiburg, Germany

**Keywords:** meniscus, osteoarthritis, mechanical examination, instantaneous modulus, Pauli score, healthy meniscus vs. arthritic

## Abstract

This study was designed to provide information on how the menisci change over the course of osteoarthritis, particularly with regard to their mechanical properties. The aim was to determine the difference between healthy menisci (fresh frozen meniscal transplants) and menisci harvested during total knee arthroplasty. The latter allows the grading of age-related and osteoarthritic changes in the menisci on macroscopic and microscopic levels. A total of 10 menisci from arthritic knee joints (medial) harvested during total knee arthroplasty were used and compared with 10 medial fresh frozen meniscal transplants. The mechanical measurements were carried out on a Mach-1 testing machine using indentation testing to determine the instantaneous modulus and the thickness of the menisci. The specimens were then embedded in paraffin, sectioned on a microtome, and stained with hematoxylin–eosin and safranin-O. All measurements were divided into the anterior horn, pars intermedia, and posterior horn. There was no significant difference in the instantaneous modulus for the posterior horn in the fresh frozen menisci with 0.27 ± 0.1 MPa compared to the arthritic menisci with 0.18 ± 0.03 MPa. No significant difference could be determined for the meniscus thicknesses. There was a significant difference in the safranin-O staining. There were also significant differences in the Pauli score: the arthrosis menisci showed a sum score that was, on average, four times higher than the sum score of the fresh frozen menisci. In the present study, it could be shown very well that there are significant differences in the mechanical properties as well as in the macroscopic and histopathological scores, such as the Pauli score, between the fresh frozen meniscus allografts considered healthy and osteoarthritic menisci resulting from total knee arthroplasty. With a degradation score of 3 (Pauli), the instantaneous modulus was reduced by more than 50% compared to healthy controls. More importantly, however, the fresh frozen menisci only show a grade 2 when converting the sum values into grades, where a grade 2 indicates slight degeneration. This is interesting because fresh frozen meniscus transplants were always considered healthy in previous publications and should, therefore, actually have a grade 1.

## Introduction

Osteoarthritis is a disease affecting the entire joint, i.e., bones, ligaments, connective tissues, and synovium, as well as associated structures such as the menisci. With a prevalence of 17% in people over 18 years of age, it is the most common joint disease in Germany (RKI, 2021), with the knee joint being the most commonly affected (Luong et al., 2012). In the initial phase, patients complain about pain after prolonged sitting and lying down, and in the subsequent phases, complaints include exacerbation of rest pain, a feeling of instability, joint blockages, crepitations, muscle atrophy, joint swelling, effusion formation, overheating, and impairment of personal and professional everyday life (Breusch and Abel, 2006). Treatment options range from conservative to minimally invasive to endoprosthetic care in cases of advanced osteoarthritis (Devos-Comby et al., 2006; Hangody and Vásárhelyi et al., 2008; Mayr and Reinhold et al., 2015). The latter, in particular, is one of the five most frequently performed surgical procedures in Germany in 2020 (Bundesamt, 2020). This represents an increasing socioeconomic burden, especially under the aspect of demographic change and the reverberant effect of the COVID-19 pandemic on our healthcare system. In addition to the classic risk factors, such as age, weight, gender, incorrect loading, trauma, and axial deformities (Niethard et al., 2009; O'Neill and McCabe et al., 2018), meniscal damage, in particular, plays a significant role in the development of gonarthrosis (Berthiaume and Raynauld et al., 2005; Blagojevic and Jinks et al., 2010). Meniscal damage can be traumatic on the one hand and degenerative on the other, and it usually requires arthroscopically assisted surgical treatment. Current surgical treatments aim to preserve or remove as little meniscal tissue as possible in order to maintain its function of load distribution and joint stabilization by balancing joint incongruence (Gilbert and Chen et al., 2014), shock absorption (Voloshin and Wosk, 1983), proprioception, joint lubrication, and nutrition. In cases of extremely degenerated or traumatically destroyed meniscal tissue, meniscal allograft transplantation aims to limit premature knee degeneration, reduce pain, and improve knee function. In recent times, meniscal allograft transplantation (MAT) has emerged as the cutting-edge treatment for orthopedic surgeons dealing with symptomatic patients who have undergone subtotal or total meniscectomy, particularly in the younger population (Trentacosta and Graham et al., 2016; Figueroa et al., 2019). So far, even fresh allograft menisci have a limited survival time due to host *versus* graft reactions (Verdonk and Demurie et al., 2005). In addition, the availability of fresh menisci is very limited, especially in Europe, due to strict legal regulations. The traditional indications for MAT involve individuals experiencing symptomatic meniscal deficiency, provided that advanced degenerative changes are not present. Additionally, any accompanying issues, like localized osteochondral defects, instability, or malalignment, should be suitable for surgical correction (Figueroa et al., 2019). In a previous study (Pordzik and Bernstein et al., 2020a), mappings of menisci were already carried out by comparing the OA and healthy control groups. Both groups were in a similar age group of 70 years. It can, therefore, be assumed that healthy menisci showed age-related damage (despite the absence of OA). For this reason, we initiated this study to make a similar comparison with a completely healthy control group in the age group of 35 years. In addition, we wanted to compare the histological results of the Pauli score and also determine the proteoglycan content, as this is an additional indicator of the integrity of the menisci. A similar study design has not been used before and, in our opinion, represents a gain in knowledge that can be used in further studies. In this study, we were also interested in whether MAT can really be considered an improvement and can be described as “healthy” menisci.

## Materials and methods

### Materials

A total of 10 menisci of arthritic knee joints (10 medial) were used, which were obtained during the implantation of a total knee arthroplasty (knee replacement) in the presence of existing subjective and confirmed native radiographic osteoarthritis according to Kellgren–Lawrence grades III–IV (Kellgren and Lawrence, 1957). The average patient’s age (female 7/male 3) was 70.6 ± 12.7 years. On the other hand, 10 medial fresh frozen meniscus allografts from JRF Ortho obtained by Neutromedics AG (Cham, Switzerland) were used as the control. The latter could not be implanted during the orthopedic procedure (different sizes are always thawed in order to achieve the best possible fit for the patient), so they were frozen again and released for use in scientific projects. In consultation with the manufacturers, a donor age of less than 35 years was ensured. All samples used in this study were handled according to the statutes of the Ethics Committee of the University of Freiburg (ethics vote: 305/10; 2009-05-10). The patients/participants provided their written informed consent to participate in this study.

### Methods

**FIGURE 1:**
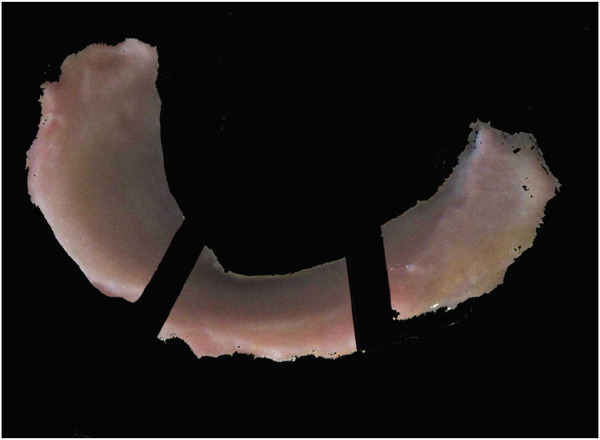
Subdivision into the anterior horn, pars intermedia, and posterior horn for mechanical and histological examinations.

The segmentation into the anterior horn, pars intermedia, and posterior horn was performed according to the preliminary work of our research group (Pordzik and Bernstein et al., 2020b) (please see Figure 1).

### Biomechanics

#### Indentation

**FIGURE 2:**
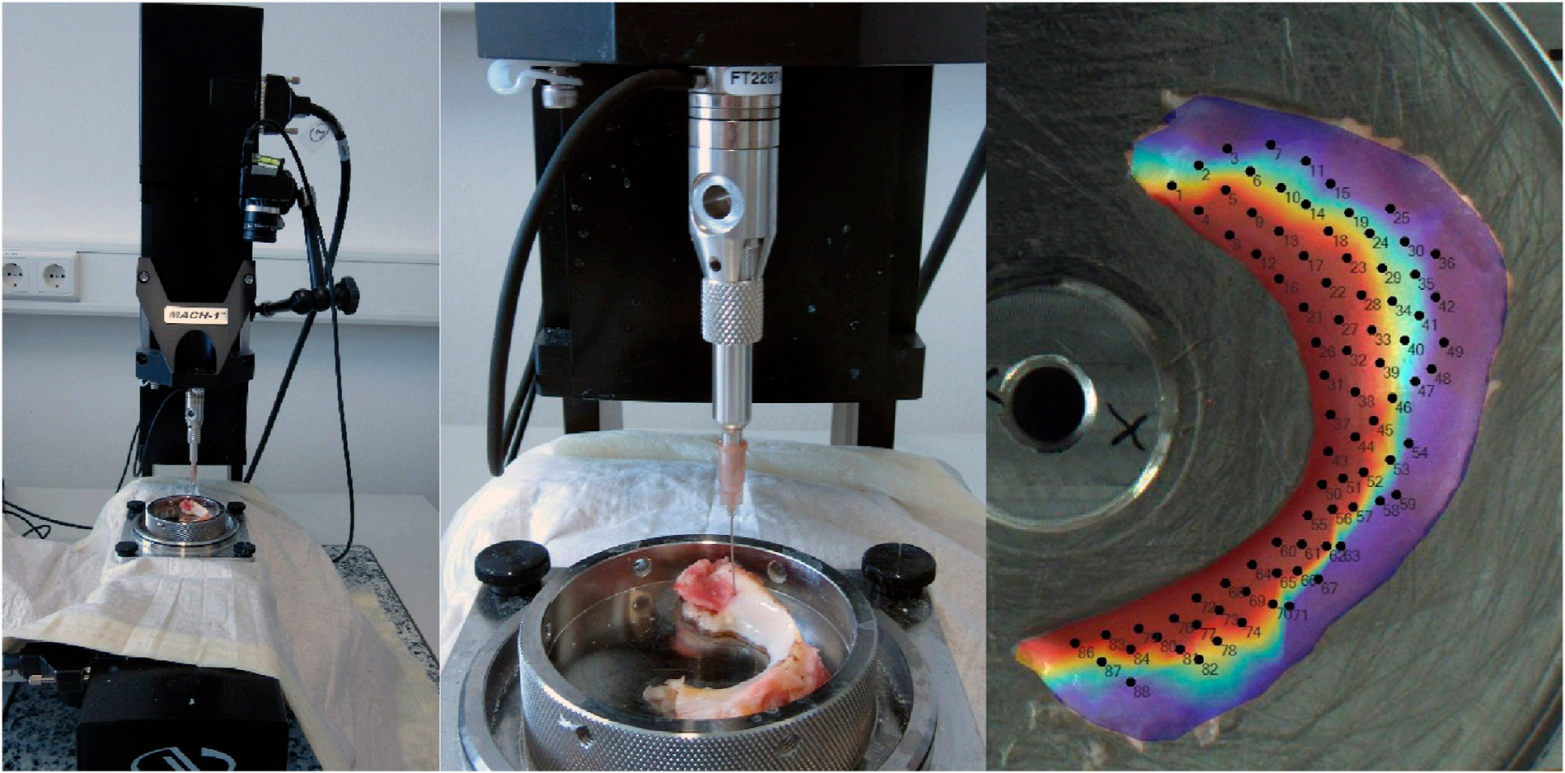
Setting Mach-1 for indentation and thickness measuring using the needle method and projection of the measurement positions on the meniscus.

The biomechanical measurements (normal indentation) were performed according to established protocols in previous studies (Seidenstuecker and Watrinet et al., 2019; Pordzik and Bernstein et al., 2020a) and following previous publications on indentation testing (McKee and Last et al., 2011; Sim and Chevrier et al., 2017). The measurements involved the use of the Mach-1 (v500css, Biomomentum Inc., Montreal, QC, Canada), a multi-axial 70 N load cell (MA23X, ATI Industrial Automation, Apex, NC, USA), the Newport Motion Controller (ESP 301, Newport, Irvine, CA, USA), a sample holder (MA646, Biomomentum Inc., Montreal, QC, Canada), and a spherical indenter with a radius of 1 mm (MA680, Biomomentum Inc., Montreal, QC, Canada). Initially, the menisci were attached to the sample holder with superglue (Henkel AG Co. KGaA) to prevent positional displacement during the measurement procedure and allow subsequent tissue-sparing defixation. During the indentation and thickness tests, the menisci were irrigated at regular intervals with 0.9% NaCl (Fresenius Medical Care) to prevent drying. The first step is to calibrate the load cell and indenter used on the Mach-1. The sample is then fixed in place. A photo of the sample is then taken exactly from above with the Mach-1, the measuring table is calibrated in the *X* and *Y* directions, and the measuring positions (at least 80 per meniscus using a scanning raster of 0.25 mm) are marked on the image of the meniscus. NaCl (0.9%) was then applied to protect the meniscus from drying out. The automatic indentation (normal indentation) test then began with a spherical indenter with an indenter radius of 0.5 mm. The *Z*-axis speed in the direction of the sample was limited to 1 mm/s. The contact criterion (contact indenter–surface) was defined as 0.1 N. The scanning raster between the measuring points on the virtual mesh was set to 0.25 mm (see Figure 2). The indentation amplitude was set to 0.2 mm, and the indentation velocity was 0.2 mm/s with a relaxation time of 10 s. To protect the load cell, the termination criterion was set to 5 N.

The instantaneous modulus (IM) was evaluated at each measuring position according to Hayes and Keer et al. (1972) using the following equation for normal indentation:

$$IM=\frac{P}{H}∙\frac{1-\vartheta^{2}}{2ak∙\left(\frac{a}{h}\vartheta \right)}.$$

Here, IM is the instantaneous modulus; P is the load; H is the depth of indentation; a is the radius of the contact region; ϑ is the Poisson´s ratio (=0.5); k is the correction factor dependent on a/h and ϑ; h is the sample thickness for normal indentation.

#### Thickness

The thickness was measured in terms of a needle technique (Jurvelin and Räsänen et al., 1995). For this purpose, the indenter was replaced by a Sterican 18G cannula (B. Braun, Melsungen, Germany). To prevent the 18G needle from bending and leading to incorrect thickness values, the needle was replaced after each measuring point. Furthermore, the following parameters were set in Mach-1 Motion Software:

- Contact criterion, 0.1 N (indenter–sample)
  - Stage velocity, 0.5 mm/s
  - Stage reposition, 2x load resolution
  - Termination criterion, 5 N (to protect the load cell)

The same scan grid was used as before for the indentation test. In this method, the distance traveled by the needle between the initial contact (0.1 N) with the meniscus tissue and the break-off contact (5 N) with the metallic base is recorded and then evaluated using Mach-1 Analysis Software. The thickness was corrected using the cosine of the angle (Sim and Chevrier et al., 2017; Veronesi and Berni et al., 2021). The angle was automatically measured for all normal indentation positions. In addition to the values for maximum force and thickness, the IM could be calculated for each defined point of the scan grid, according to Hayes Keer et al. (1972).

### Histology

After biomechanical measurement, the specimens were refrozen and stored at −80°C until histological processing. After thawing, the menisci were divided into three parts: pars anterior (PA), pars intermedia (PI), and pars posterior (PP). A smaller portion of each third was excised in the sagittal plane for further processing. Histological preparation followed the classic triad of formalin immersion for 24 h at −4°C, dehydration in the ascending alcohol series, and paraffinization using an automatic tissue infiltration machine (Leica TP1020, Wetzlar, Germany). The 2-µm-thick sections prepared with a rotation microtome (Leica RM2255) were stained by both hematoxylin–eosin (HE) and safranin-O (SO) after subsequent rehydration in the descending alcohol series. HE staining was used to evaluate the quality of the tissue as a microscopic part of the Pauli score. SO staining was performed according to a modified protocol based on the literature (Mulisch and Welsch, 2015) and was used to evaluate the cell morphology/extracellular matrix and quantify the proteoglycan content.

I. Deparaffinize and hydrogenate: 1) 3 × 10 min xylene; 2) 10 min EtOH 100%; 3) 10 min EtOH 96%; 4) 10 min EtOH 96%; 5) 10 min EtOH 70%; and 6) 5 min bidest H_2_O
II. 10 min of Weigert’s ferric hematoxylin (50% iron hematoxylin A+ 50% iron hematoxylin B)
III. 10 min H_2_O
IV. 5 min 0.05% fast green (0.5 g fast green in 1 L bidest H_2_O)
V. 12 sec 1% acetic acid
VI. 5 min 0.1% SO (SO 0.1 g in 1 L bidest H_2_O)
VII. Immerse bidest water 2x briefly
VIII. Dehydrate:

1) Immerse EtOH 70% 2x briefly; 2) immerse EtOH 96% 3x briefly; 3) immerse EtOH 100% 5x briefly; 4) immerse EtOH 100% 10x briefly; 5) 3 min xylene, 6) 3 min xylene.

IX. Fix with Entellan on a microscope slide

For histological quantitative analysis of the proteoglycan content, the image was first digitized using a microscope (BX53 light, Olympus, Shinjuku, Japan) and a scanning stage (SCAN 130 × 85, Maerzhaeuser Wetzlar GmbH Co. KG, Wetzlar, Germany). The scanning stage and Stream Motion software 1.9.4 from OLYMPUS enable complete digitization of the meniscus section at 10 × magnification with the same exposure by automatically stitching together several individual images.

### Quantitative evaluation of the proteoglycan content

The quantitative evaluation of the proteoglycan content based on SO staining was performed by image processing using FIJI by ImageJ 1.53q (Wayne Rasband and contributors, National Institutes of Health, USA). In the first step, the region of interest was defined using the “Wall (tracing) tool” at a tolerance strength between 6 and 9, its area was calculated in terms of pixels using “Ctrl + M,″ and the surrounding background was removed using “Clear Outside.” In the following step, all meniscus parts stained with Fast Green were selected using “Color Threshold” (hue 200–255, saturation 0–255, and brightness 0–255), and their removal was done using “Clear.” In the final step, the resulting image was reopened, and “Color Threshold” (hue 0–255, saturation 0–255, and brightness 50–255) was used to select all SO-stained parts and determine their area again using “Ctrl + M.” The calculated area was finally set in relation to the ROI. Additionally, the color intensity of the SO-stained portion was determined on a scale of 0–255 using the macro “Ctrl + H.”

For histological qualitative analysis of the menisci, both macroscopic and microscopic grading was performed according to the scoring system of Pauli, Grogan et al. (2011). In addition, microscopic evaluation of the surface (divided into femoral, tibial, and inner edge), cellularity, collagen organization, and SO staining were performed for all three parts (anterior horn, pars intermedia, and posterior horn).

### Statistics

Statistical analysis was performed using Microsoft Excel 2019 (Microsoft Corporation, Redmond, WA, USA) and Origin 2022SR1 (OriginLab Corporation, Northampton, MA, USA). All values were expressed as the mean ± standard error of the mean. Regarding the scores and all numerical values (if n < 5), statistical significance was tested non-parametrically, primarily using the Mann–Whitney *U* test. Probability distributions of the samples (n ≥ 5) were analyzed using the Kolmogorov–Smirnov test and the Pearson-rho correlation test to determine possible influencing factors, such as meniscus thickness, SO%, and macroscopic or microscopic Pauli score, on the mechanical properties. Statistical significance was defined as *p* < 0.05. Based on the number of samples, it can be assumed that the samples are not normally distributed.

## Results

All measurements were grouped into the anterior horn, pars intermedia, and posterior horn to compare the menisci derived from total knee replacement (hereafter referred to as KR-M) with fresh frozen meniscus allografts (hereafter referred to as FF-M).

**FIGURE 3:**
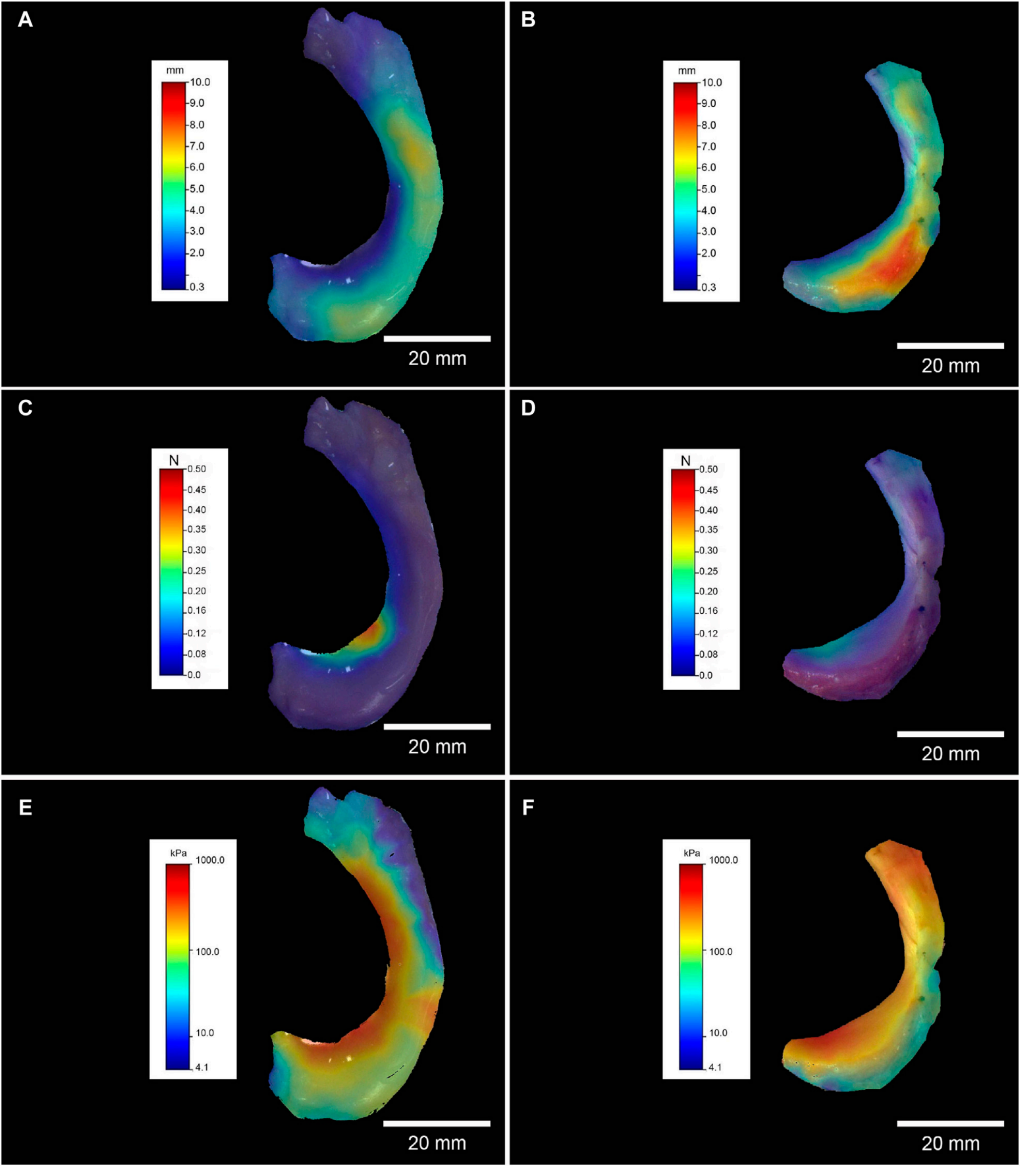
Left column, FF-M; right column, KR-M. Exemplary mapping of the thickness in mm for **(A, B)**, F max in N for **(C, D),** and IM in kPa for **(E, F)**; all mappings represent medial menisci.

The exemplary overview in Figure 3 of the thickness shows similar height values between 3 and 4 mm for both kinds of menisci (FF-M and KR-M). The maximum force and IM mapping already show that the arthritic menisci are significantly firmer than the healthy ones. While more than 100 kPa are indicated only in the inner marginal area of the IM of FF-M, almost the entire meniscus of the knee replacement group shows values clearly above 100 kPa (Figure 3F).

## Biomechanics

### Instantaneous modulus

**FIGURE 4:**
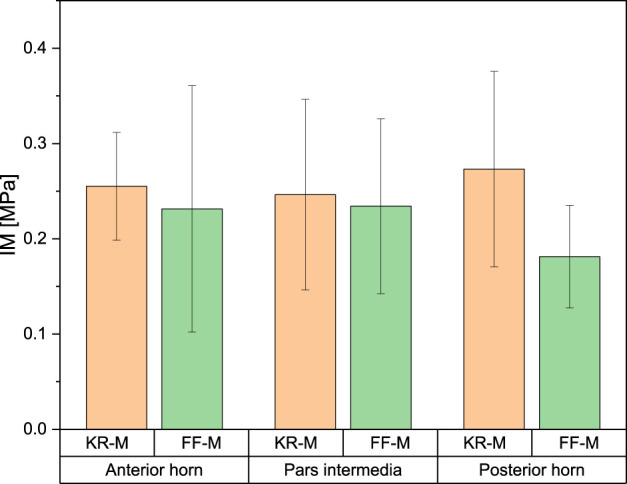
Overview of the IM by origin and meniscus subarea.

For the IM, the mean values for the anterior horn were 0.23 ± 0.09 MPa for FF-M and 0.27 ± 0.06 MPa for the menisci derived from knee replacement. This difference was not statistically significant. The IM for the pars intermedia had a mean value of 0.234 ± 0.06 MPa for FF-M and 0.234 ± 0.10 MPa for KR-M. For the posterior horn, the mean values were 0.181 ± 0.03 MPa for FF-M and 0.273 ± 0.15 MPa for KR-M. The differences in mean values were not statistically significant at *p* < 0.05. Figure 4 summarizes the results.

### Meniscus thickness

**FIGURE 5:**
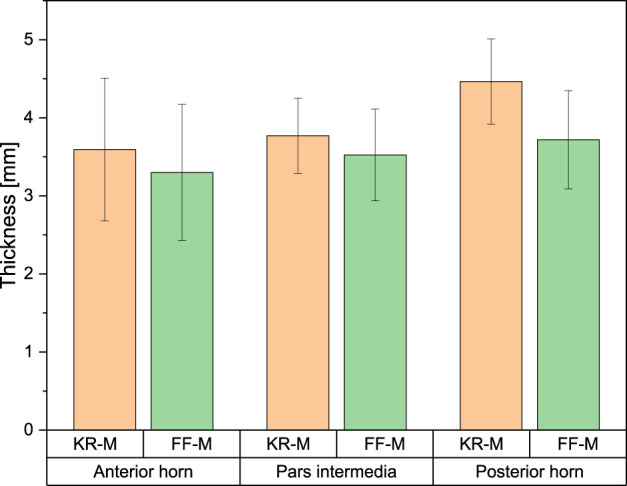
Overview of meniscus thickness for different meniscus compartments and origins.

The measured values of meniscus thickness ranged from 3.3 ± 0.6 mm for the anterior horn of FF-M and 4.14 ± 0.66 mm for the posterior horn of KR-M. There was no significant difference across all measured values with *p* < 0.05 (see Figure 5).

### Histology

#### Safranin-O staining

**FIGURE 6:**
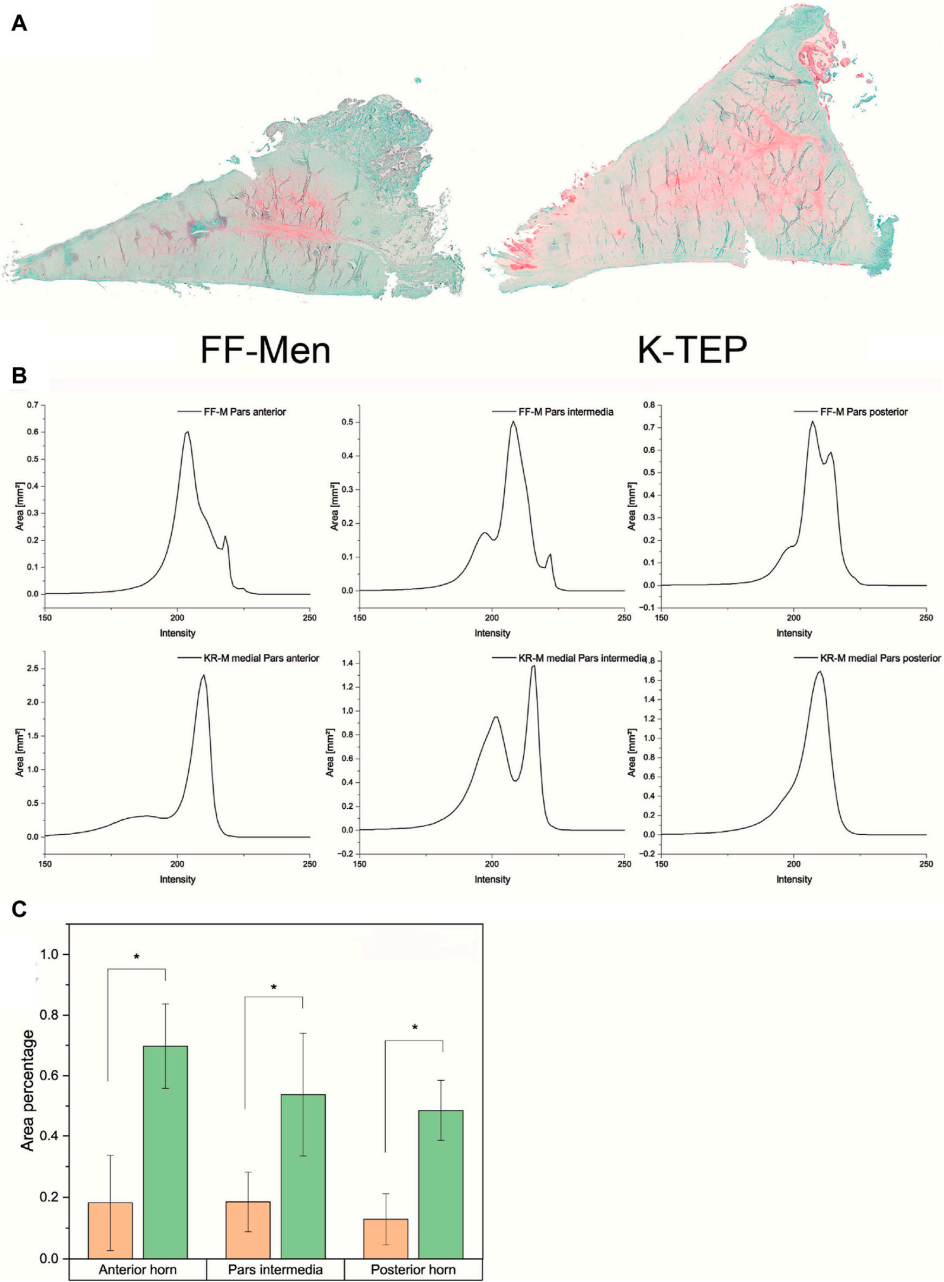
**(A)** Comparison of the proteoglycan content of menisci from different origins using safranin-O staining. **(B)** Overview of the color intensity curves and SO-stained proportions of the total area of the meniscus for the menisci of different origins. **(C)** Comparison of area fractions for meniscus compartments of different origins. Significant differences are indicated with *p* < 0.05 (*).

Light microscopy revealed a difference in the safranin-O staining of the menisci of different origins. The knee replacement menisci showed significantly more intense staining, as well as a significantly larger stained area and, thus, a significantly higher proportion of proteoglycan. Figure 6A shows examples of safranin-O-stained knee replacement and FF-M, both images taken with an exposure time of 10 ms. The color intensity curves and percentages of the total meniscus area are shown in Figure 6B. From the intensity values, the SO-stained areas were then related to the total meniscus area resulting for the anterior horn, which was 18.2% for FF-M and 69.8% for KR-M, i.e., a tripling of the proteoglycan content. Similar results were also found for the pars intermedia, with 18.5% vs. 54% (FF-M vs. KR-M) and 12.9% vs. 48.5% for the posterior horn. An overview of the area fractions and thus the proteoglycan content is shown in Figure 6C.

#### Pauli score

**FIGURE 7:**
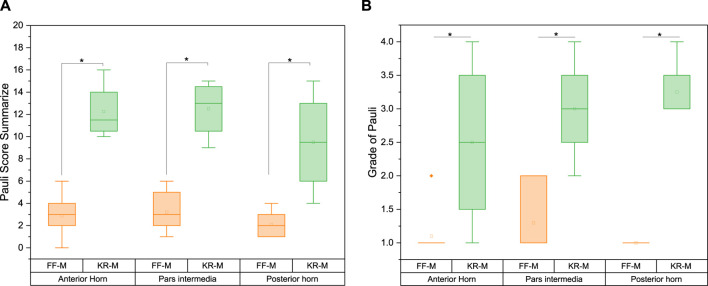
Pauli score **(A)** sum and **(B)** grade for the menisci of different origins. Significant differences are indicated with *p* < 0.05 (*). Grade 1 represents normal tissue, grade 2 indicates mild degeneration, grade 3 indicates moderate degeneration, and grade 4 indicates severe degeneration.

There was a significant difference in the Pauli score between FF-M and KR-M with *p* < 0.05. The sums of the Pauli scores for the KR-M were, on average, four times greater than those of the FF-M, irrespective of the meniscus section (Figure 7). Converting the sum scores into grades (Pauli, Grogan et al., 2011) with G1 = 0–4, G2 = 5–9, G3 = 10–14, and G4 = 15–18 results in Figure 7B.

On average, the recalculated grade from the Pauli score for the sections of the KR-M is approximately twice as high as for the FF-M (Figure 7). The differences between the sections of the menisci are significant with *p* > 0.05. As expected, healthy menisci show a grade of 1, while osteoarthritic menisci show a grade of 3 (moderate degeneration).

#### Pearson correlation

**FIGURE 8:**
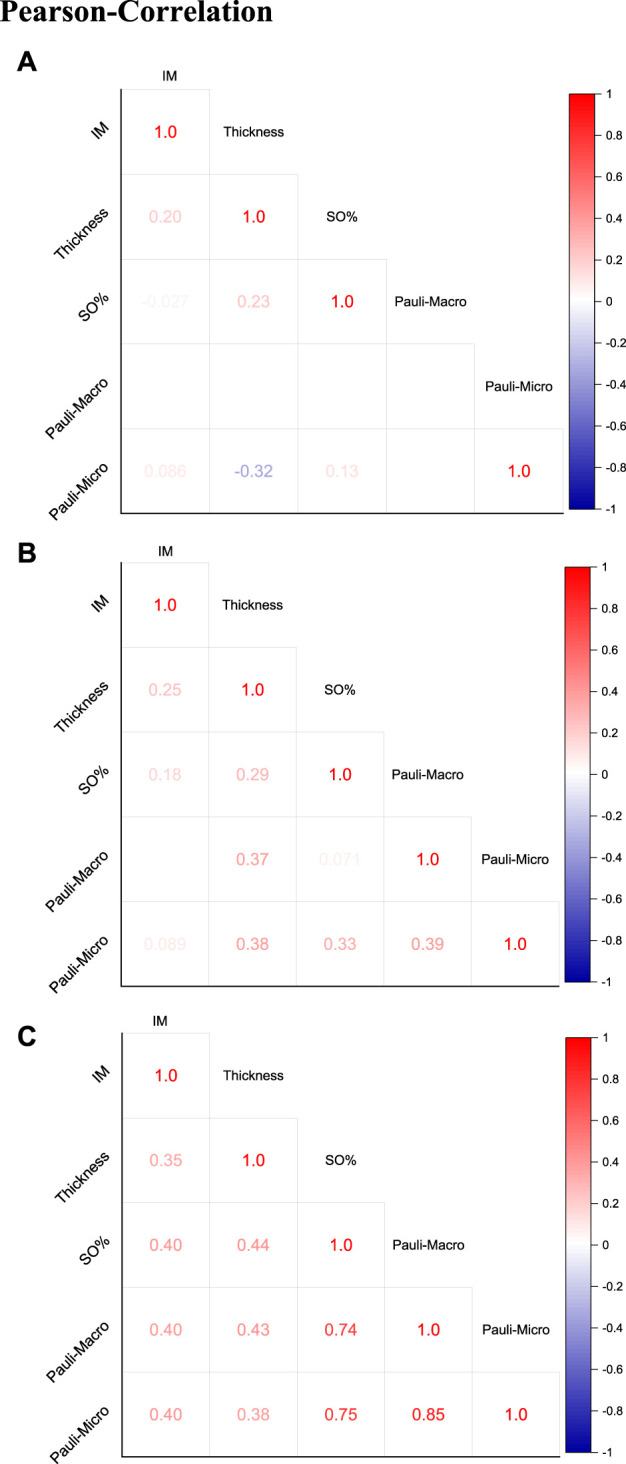
Pearson correlation with presentation of the correlation coefficients for **(A)** FF-M, **(B)** KR-M, and **(C)** all samples (KR-M and FF-M).

The overview of the Pearson correlation matrices in Figure 8 summarizes the matrices for the FF-M (Figure 8A), KR-M (Figure 8B), and all samples (Figure 8C). The correlation of all samples (Figure 8C) showed a strong positive correlation in the Pauli microscopy and macroscopy as well as between SO% and the Pauli macroscopy/microscopy. A weak to medium positive linear correlation was found between the IM and all other parameters and for the correlation between thickness and all other parameters.

## Discussion

The mapping of the IM showed a similar picture as already published in a paper by us (Pordzik and Bernstein et al., 2020b). However, that paper compared two groups with similar ages: 72 ± 7 years for the arthritic group vs. 70 ± 5 years for the arthritis-free group, whereas in the present study, the age of the FF-M donors was less than 35 years and that of the KR-M donors was 70.6 + 12.7 years. It could also be assumed that the healthy control group in Pordzik and Bernstein et al. (2020a) exhibited age-related damage to the meniscus. The repeated thawing and freezing of the FFM for the operations in the present study, in which the menisci were then not used due to their size, may also have resulted in meniscal damage, which is expressed by the reduced Pauli grade.

Looking at the mean values of the IM for the meniscus compartments, the highest values were observed in the area of the posterior horn. This is not surprising, as the posterior horn is the most stressed area. The increase in the IM in the menisci originating from the knee replacement suggests a calcification of the meniscus. Very few previous studies have fully mapped human menisci (Pordzik and Bernstein et al., 2020b; Seitz and Osthaus et al., 2021), with most focusing only on punched-out areas (Sweigart and Zhu et al., 2004; Katsuragawa and Saitoh et al., 2010; Seitz and Galbusera et al., 2013). Only Danso and Mäkelä et al. (2015) attempted indentation tests with menisci without destroying them, but the number of measurements along the meniscus was three, which is too few to make valid statements. Sim and Chevrier et al. (2017) used automatic mapping through Mach-1, but unfortunately, the study was not on menisci but on the human distal femur and tibia plateaus. Compared with the values measured in the previous study (Pordzik and Bernstein et al., 2020b), however, there are still differences: the values for the knee replacement are higher at 0.25–0.4 MPa than in the prior study (0.17 ± 0.07 MPa). The values of the healthy control, this time even in the age range of 35 years, also showed increased values of 0.18–0.24 MPa compared to Pordzik and Bernstein et al. (2020a). However, this may be due to the age difference and associated structural changes within the meniscus (Tsujii and Nakamura et al., 2017). Nakano and, Dodd et al. (1997) described a decrease with aging in fibronectin, a glycoprotein that enhances molecular adhesion in the ECM; it has been found to contribute to cartilage fibrillation. This may be responsible for the difference between IM of the two “healthy” controls. Seitz and Osthaus et al. (2021) determined similar measured values for the IM for AH, PI, and PH for medial menisci, with values between 0.2 and 0.3 MPa for mild degeneration and even higher values for severe degeneration than in our measurements with 0.2–0.6 MPa. They varied the type of fixation on the specimen holder by using a cast of the meniscus to mimic the articular cartilage. The meniscus was fixed with ligaments as in the knee and could slide laterally as in the knee. In addition, they used the same testing machine as in the present study: a Mach-1 CSS. Compared to our measurement, they used a larger indenter diameter of 2 instead of 1 mm, deeper indentation depth (0.5 vs 0.2 mm for us), and greater indentation speed (0.5 vs 0.2 mm/s) as in the present study, which may have led to different values.

Bursac and Arnoczky et al. (2009) and Danso and Oinas et al. (2017) described the same relationship: an increase in compression modulus with a concomitant increase in the glycosaminoglycan content. Kwok and Grogan et al. (2014) described an increase in stiffness with an increase in the proteoglycan content.

Even the fresh frozen menisci show certain dehydration. This influences the volume and mechanical properties (Gelber and Gonzalez et al., 2008; Lee and Chung et al., 2012). Lee and Chung et al. (2012) described a slight to moderate shrinkage of the implanted meniscus (replacement) over a period of 10 years. Nevertheless, there is already a loss of fluid due to shock freezing. This is supported by our results regarding the area percentage of proteoglycan as an indicator of the water content of the meniscus. It is known that the osteoarthritic meniscus increases in the water content (Peters and Smillie, 1972; Kwok and Grogan et al., 2014; Pordzik and Bernstein et al., 2020a; Seitz and Osthaus et al., 2021). The already low water content of the healthy menisci was further reduced by shock freezing, which led to a significant difference in the proteoglycan content compared to the osteoarthritic meniscus.

The healthy (freshly frozen) menisci show a very low Pauli sum score, which corresponds to 1 (healthy tissue) when converted into the degeneration grades introduced by Pauli and Grogan et al. (2011). In contrast, the osteoarthritic menisci (KR-M) are in the upper range of the Pauli sum score, with values between 12 and 16. The conversion to the degree of degeneration results in a value of 3, which corresponds to moderate degeneration.

### Limitations

When considering the reliability of the results of the current study, several factors must be taken into account. First, we chose medial menisci due to biomechanical concerns (the main type of loading). The results cannot, of course, be applied to lateral menisci. Second, the preparation technique, in particular, the bonding of the menisci to the specimen holder with Loctite, may have influenced the biomechanical measurements. Looking at the mappings for Fmax, there are individual measurement points in the thin edge area. However, their share is less than 2% of the total number of measured positions, so the influence can be regarded as rather marginal. The menisci were kept moist during the measurements, but it cannot be ruled out that the menisci lost fluid and that this could have influenced the measurements. The FF-M, in particular, is known to lose fluid during thawing. Similar to other *in vitro* indentation tests with cadaveric specimens, this approach cannot replicate the complex loading behavior of the knee joint, especially the circumferential stress that occurs during physiological loading. Consequently, the indentation properties of the menisci determined in this study do not represent the properties of the entire tissue but are primarily influenced by the superficial layer and the locally defined area around the indenter. In addition, the healthy samples are freshly frozen menisci, which exhibit an increased degree of dehydration due to shock freezing, potentially affecting the measurements. Despite these considerations, a remarkably high reproducibility (>97%) of the determined biomechanical properties was achieved through extensive pre-testing, including trials on various Mach-1 testing machines. The Mach-1 testing machine settings for biomechanical mapping by indentation, including indentation depth, velocity, and relaxation time, were not based on physiological characteristics such as stresses and strains. Instead, these parameters were taken from a previous study (Sim and Chevrier et al., 2017) to ensure comparability with the existing literature. Both in the preliminary experiments and previous studies on human menisci, we identified the amplitude and speed of indentation as the most critical parameters for achieving convergence. In addition, the anatomical sub-regions were determined using a standardized method that does not take individual anatomy into account. Beyond that, we are currently working with Andreas Seitz on a publication on the influence of the fixation of the menisci on the sample plate on the resulting mechanical properties, which will also be submitted as part of this special issue. In the current setting of Mach-1 software, the minimization of the influence of the substrate is not provided, especially if you can only enter distances. We are working together with the manufacturer to be able to specify the compaction of the meniscus instead of the distance (if depending on the thickness, then always 10 or 20%).

### Conclusion and outlook

In the present study, which compared the properties of medial fresh frozen vs. medial arthritic menisci, it could be shown very well that there were no significant differences in the mechanical properties but in the macroscopic and histopathological scores, such as the Pauli score used, between the medial fresh frozen meniscal transplants (which were considered healthy) and arthritic medial menisci after total knee arthroplasty. At a degradation score of 3 (Pauli), the instantaneous modulus was reduced by more than 50% compared to healthy controls. In future studies, one could consider including mechanical properties as a parameter in a modified Pauli score, but this would require medial and lateral fresh frozen meniscal allografts. The results of our study showed that fresh frozen menisci should not be regarded as healthy; in particular, the damage caused by shock freezing cannot be denied.

## Data Availability

The raw data supporting the conclusion of this article will be made available by the authors, without undue reservation.
